# *In situ* post-ischemic conditioning by temporary balloon occlusion for acute ischemic stroke: a modified reperfusion technique and adverse event monitoring

**DOI:** 10.3389/fneur.2025.1634885

**Published:** 2025-10-02

**Authors:** Sifei Wang, Shuai Liu, Bohao Zhang, Xiao Jiang, Ming Wei, Hua Yan

**Affiliations:** ^1^Huanhu Hospital Affiliated to Tianjin Medical University, Tianjin Medical University, Tianjin, China; ^2^Tianjin Key Laboratory of Cerebral Blood Flow Reconstruction and Head and Neck Tumor New Technology Translation, Tianjin Neurosurgical Institute, Tianjin Huanhu Hospital, Tianjin, China; ^3^Department of Neurosurgery, Tianjin Huanhu Hospital, Tianjin Medical University, Tianjin, China

**Keywords:** modified reperfusion technique, acute ischemic stroke, large vessel occlusion, endovascular therapy, neuroprotective

## Abstract

**Background:**

With the development of endovascular therapy, the success rate of reperfusion for emergent large vessel occlusion (LVO) has significantly improved, but more achievements are still needed. *In situ* post-ischemic conditioning (IPC) is a modified reperfusion technique and a neuroprotective method. It is potentially promising for patients with acute ischemic stroke (AIS) and can improve the outcome; however, the IPC technique has not been well defined.

**Methods:**

The definition and technical details of *in situ* post-ischemic conditioning using neurointerventional strategies were defined in this prospective cohort. Consecutive patients treated with the IPC technique between January 1, 2022, and Jun 30, 2023, were included in this study. Patients’ demographic and technical performance of the IPC procedure were recorded. Adverse events related to the IPC procedure were actively monitored and analyzed.

**Results:**

In total, 40 patients underwent IPC. Their mean age was 65 years (IQR, 34–83), and 72.5% were male. The median ASPECTS (Alberta Stroke Program Early CT Score) was 7. The median of NIHSS was 13. The preset IPC program was achieved in 95.0% of cases, with 0% procedure-related mortality and 2.5% morbidity. The incidence of procedure-related dissection was 0%. The incidence of thrombotic events was 2.5%. Extravasation of contrast media was recorded as a serious adverse event. One thrombotic event occurred in the LAA Group. However, there was no statistical difference in the incidence of adverse events between the LAA and non-LAA groups. The LAA and MCA groups had shorter IPC procedure times compared with non-LAA and ICA groups.

**Conclusion:**

The IPC technique is safe and feasible with an acceptable complication rate.

## Introduction

Endovascular treatment has been widely used for reperfusion therapy in AIS the success rate of reperfusion treatment is high, but the independence rate does not meet the expectations (1). Blood flow reperfusion brings beneficial effects but can also damage the nervous system. Complex mechanisms, such as excitatory amino acids, oxygen free radicals, and calcium overload, are involved in the pathogenesis of ischemic reperfusion injury, also known as ischemia/reperfusion injury (I/R) (2). Post-ischemic conditioning is a modified reperfusion method. After reperfusion, brief reperfusion interruptions can play a neuroprotective role. This strategy has been verified in experimental animal models. Intermittent reperfusion and occlusion reduced infarct volume, relieved brain edema, and improved nervous function in the rat model of I/R (3, 4). The remote ischemic conditioning (RIC) technique has been extensively applied in clinical treatment of patients with AIS. This method targets extracranial arteries around the upper (or lower) extremity (5, 6). Compared with RIC, the IPC technique has not been established in the intracranial blood vessels for AIS patients. A reperfusion technique with cycles of inflation/deflation can be performed using a percutaneous transluminal angioplasty balloon during primary percutaneous coronary intervention (PCI) for STEMI (7, 8). The endovascular treatment of AIS can mirror the operation in the human body similar to PCI. Intermittent occlusion for post-ischemic conditioning can be done by targeting the affected blood vessel, known as IPC. In this cohort, IPC was clearly defined and used to guide clinical application. At the same time, strict active adverse events monitoring procedures were set up. In addition, we discussed the effect of etiology and occlusion site on the efficacy of IPC.

### Study population

This ongoing, prospective, single center cohort study in China recruited eligible patients consecutively from Tianjin Huanhu Hospital between January 1,2022 and June 30,2023 and the study was registered as Ischemic Post-conditioning in the Treatment of Acute Ischemic Stroke (IPC-TRACK, NCT06456437) linked to the triage registry of large vessel occlusion (TRACK-LVO, NCT0565916). The institutional review board and ethics committee approved the study protocol. Specific inclusion and exclusion criteria were adopted in PROTECT protocol (9). Inclusion Criteria:(1) Ischemic stroke confirmed by CT or MRI of the head; (2) Large vessel occlusion confirmed by CTA or MRA of the head, including intracranial internal carotid artery (ICA), middle cerebral artery (MCA M1/M2); (3) Recanalization of the occluded vessel at eTICI grade 2b/3 as confirmed by DSA after thrombectomy. Exclusion Criteria: (1) inability to perform an MRI or CT scan for any reason; (2) the patient has any condition that would interfere with neurologic assessment or psychiatric disorders; (3) stroke onset with seizures resulted in the inability to obtain an accurate NIHSS baseline; (4) pregnancy; (5) other serious, advanced or terminal illness; (6) moderate or severe residual stenosis (≥ 50%) of the culprit artery after recanalization; (7) difficulty in complying with ischemic post-conditioning or other conditions that the investigator considered inappropriate for inclusion.

Ethics approval was obtained from the ethics committee of Tianjin Huanhu Hospital. Informed consent was provided by all patients or their legally authorized representatives before participation in the study. This study was performed following the Code of Medical Ethics of the World Medical Association (Declaration of Helsinki, 2014).

### The definition of IPC for intervention

Post-ischemic conditioning as a neuroprotective method can be performed immediatlly following achieving blood flow reperfusion. It can be divided into distant and original site post-ischemic conditioning. Remote ischemic conditioning refers to the cycle of ischemia and reperfusion applied to a distant site to exert neuroprotective effects on the brain (10). Compared with remote ischemic conditioning, IPC targets the brain where acute large vessel occlusion occurs due to thrombosis or other causes. This method is defined as IPC, which can be characterized by intermittent artery occlusion by a balloon catheter (7, 8, 11). This technology does not affect non-infarct foci in the brain or other tissues. Thus, it is known as the local or direct ischemia conditioning technique. Specifically, IPC can be applied immediately after the acute reopening of great blood vessels. A balloon catheter will be placed in the affected segment of the intracranial artery where the thrombus exists.

### Principle of *in situ* post-ischemic conditioning therapy

**Figure 1:**
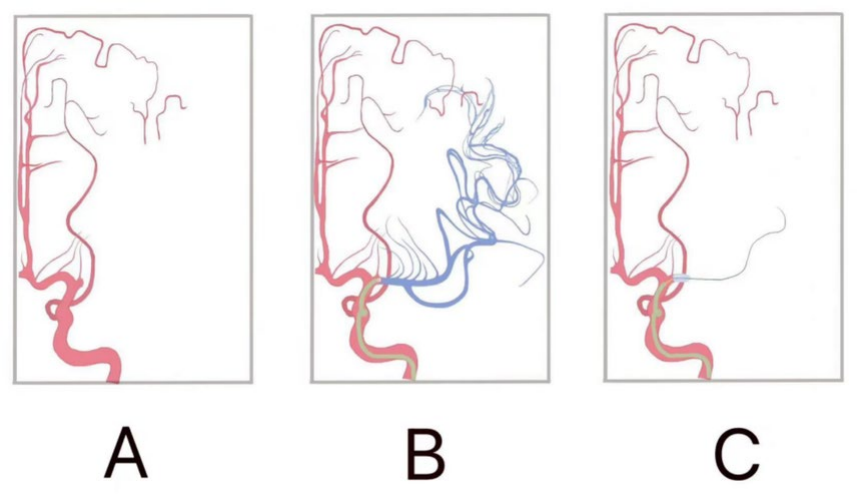
Schematic diagram of postadaptive treatment. **(A)** Occlusion of the M1 segment of the middle cerebral artery. In the red area, the anterior cerebral artery, the posterior cerebral artery, the proximal lenticulostriate artery, and the leptomeningeal anastomosis. Collateral blood flow was not involved. **(B)** Blue area, middle cerebral artery was fully open. **(C)** IPC, with the balloon placed at the occlusion site for temporary occlusion. Blue area: flow pattern was comparable to that before the surgery.

Ischemic conditioning by temporary occlusion of intracranial blood vessels has been proven to be neuroprotective (3, 4, 7). We adopted the following principles to validate this concept and decrease the potential risk of neuronal injury associated with temporary hemodynamic changes:

1) The condition therapy was performed by balloon occlusion of the affected blood vessel, and anterograde intracranial blood flow was completely blocked.
2) Balloon occlusion was treated without expanding ischemia compared with vascular occlusion before thrombectomy. The occluded vessel was at the proper site and level to simulate acute great vessel occlusion. After blocking blood flow, cerebral angiography displayed that intracranial blood flow was equivalent to control cerebral angiography before thrombectomy (Figure 1).

### DSA screening methods

To comply with the above principles, the M1 segment of the middle cerebral artery was selected as our therapeutic target. The included patients had occlusion of the M1 segment of the middle cerebral artery concomitant with the occlusion of the internal carotid artery at different segments. For different segments of the middle cerebral artery and internal carotid artery, we adopted different surgical strategies. M1 and internal carotid artery-specific classification standards were as follows: The M1 segment of the middle cerebral artery originated from the distal bifurcation of the carotid artery and extended to the first major branch of the middle cerebral artery. The ICA segmentation used for occlusion location was defined according to previous studies, giving rise to the cervical/extracranial segment (ExICA), intrapetrous plus intracavernous segment (IICA), and supraclinoid segment (SICA) (12).

The cervical/extracranial segment (ExICA), intrapetrous plus intracavernous segment (IICA), and supraclinoid segment (SICA) were all included in our study. For patients with intracranial occlusion of the internal carotid artery, we chose the middle cerebral artery without obvious anterograde blood flow on the ipsilateral side of the target lesion. In patients with the circle of Willis, the flow from the internal carotid artery contralateral to the lesion could pass through the anterior communicating artery and compensate for the middle cerebral artery on the affected side. These patients were excluded. Primary collaterals through the posterior communicating artery or secondary collaterals providing blood flow to the middle cerebral artery in ischemic regions were also excluded. Selected patients with carotid artery occlusion had tandem lesions, which means simultaneous involvement of the middle cerebral artery on the affected side. For thrombus at the distal carotid artery, the middle cerebral artery was not involved alone, but T-type or Ll-type occlusion involving the anterior cerebral artery or distal carotid artery was also included in this study.

### Endovascular techniques

The modified reperfusion technique required repetitive occlusion of the parent blood vessels in the original position of the target lesion, in which the thrombus caused ischemic stroke. At the same time, our balloon occlusion could not affect potential collaterals and normal blood vessels. Balloon occlusion generally commenced after restoring antegrade perfusion. IPC consisted of four cycles, each involving 2 min of low-pressure balloon inflation (<4 atm) followed by 2 min of deflation (Supplementary Figure 1). After completing the first episode of IPC, angiography was conducted to confirm the patency of injured blood vessels. After confirming the absence of abnormality, the second episode of IPC was performed. Next, an alternative cycle of inflation and deflation with different durations was performed. Angiography was performed after completing the IPC technique.

Balloon placement is critical for the successful implementation of IPC. In the M1 segment of the middle cerebral artery, balloon occlusion was completed after full recanalization. We chose the low pressure of semi-compliance and rapid exchange of balloons in the PROTECT cohort. This balloon was placed in arteries with complete occlusion in the control angiography before thrombectomy. We saved the main branch of the lenticulostriate artery, anterior choroidal artery, and anterior cerebral artery if patients with only middle cerebral artery occlusion did not show anterior cerebral artery or distal internal carotid artery occlusion. T-shaped or l-shaped clots at the distal of the carotid artery can override the terminal and anterior cerebral arteries. The second consideration was the size of the balloon. A balloon with the shortest length and minimum diameter was used to prevent additional damage (13). The third consideration was the pressure and inflation process. Low-pressure inflation was first used, and this pressure was the minimum pressure enough to block blood flow. When the contrast medium filled both ends of the balloon, angiography was performed to confirm arterial occlusion (7, 14).

**Figure 2:**
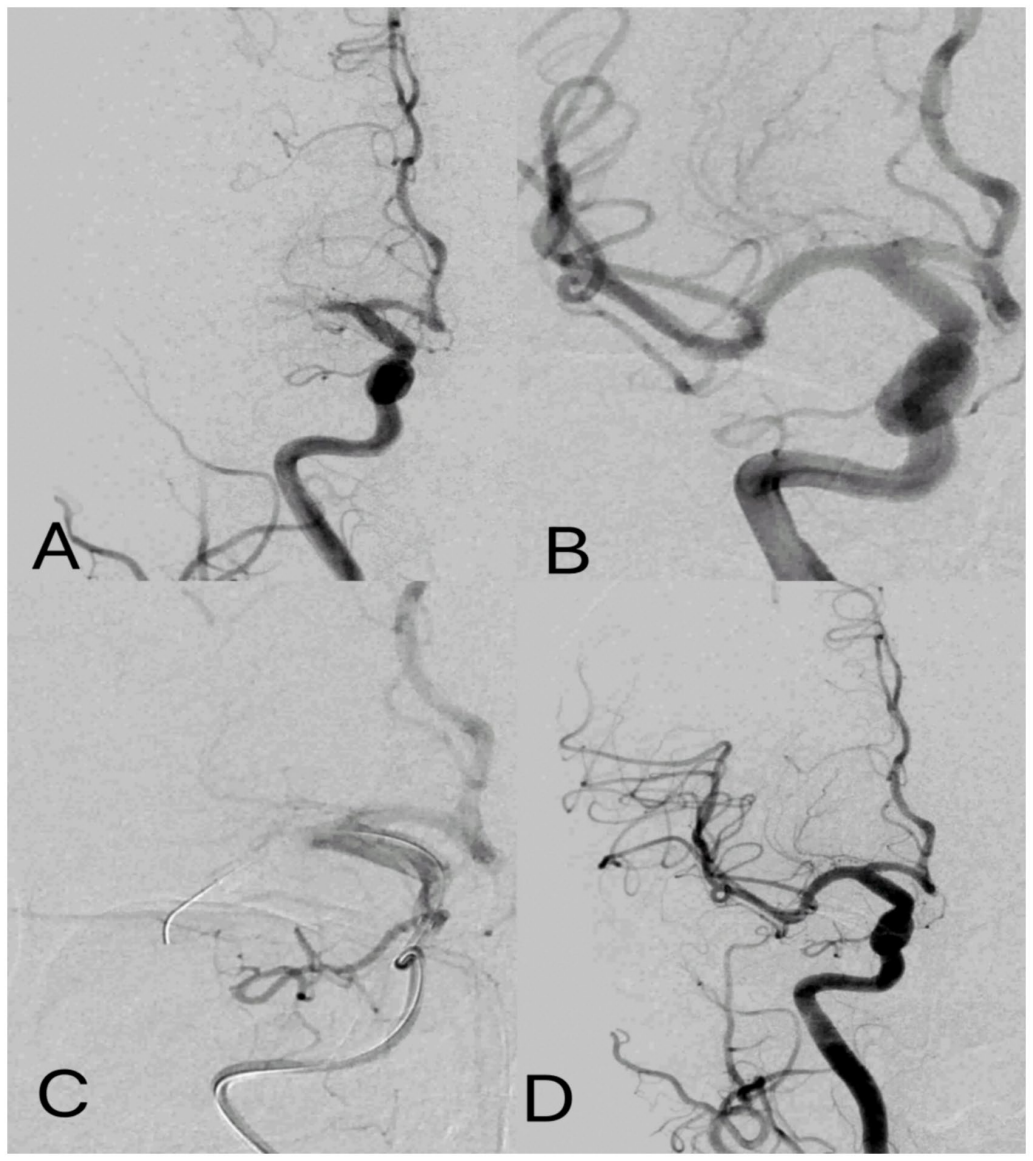
Post-ischemic conditioning of middle cerebral artery occlusion. Digital subtraction angiography: **(A)** occlusion of the M1 segment of the middle cerebral artery. **(B)** cerebral artery thrombectomy was completed, and the middle cerebral artery was fully open. **(C)** For IPC, the balloon was placed at the occlusion site to induce temporary occlusion, and angiography showed complete occlusion of the middle cerebral artery with no blood flow passing through. **(D)** IPC was completed with flow patterns consistent with those after cerebral artery thrombectomy.

The procedure was more complex for patients with internal carotid artery occlusion. Intermediate catheters, or microcatheters passed the primary lesion of internal carotid artery, and then reached the distal segment of the middle cerebral artery to achieve middle cerebral artery recanalization. Subsequently, IPC was performed. Carotid artery recanalization finished after IPC. All tandem lesion-related interventional techniques were done according to the specific situations (see Figure 2).

### Adverse event monitoring

We set up a high-quality procedure. Before the study, all researchers understood the definition, principles, and surgical procedures of IPC through text and schematics. A physician with 10 years of neurointervention experience (M.W.) guided two surgeons with 2 years of neurointervention experience (S.W. and L.L.) to identify suitable patients for the endovascular procedures according to the definition of IPC and monitored the preset endovascular procedures at the angio suite or through telephone or network. In this cohort study, adverse events were prospectively monitored. Surgeons actively monitored adverse events in the form of a questionnaire. They monitored dissection, perforation, thrombotic events, intraoperative and perioperative re-occlusion. Relevant technical performance data, such as the procedure time, device selection, placement position, and the number of balloons, were prospectively collected. An independent Data and Safety Monitoring Board (DMSB) meeting was held every six months to monitor and review adverse events.

### Statistical analysis

After the normality test (Kolmogorov Smirnoff test), statistical analyses were performed using the unpaired t test for continuous variables. χ^2^ test was used for categorical variables. All *p* values were 2-tailed, and the level of statistical significance was *p* ≤ 0.05. All statistical analyses were conducted using SPSS for Windows version 17.0 software (SPSS Inc.).

## Results

From January 1,2022 to June 30,2023, a total of 45 patients were recruited across our stroke center (Supplementary Figure 2) from IPC-TRACK cohort 0.5 patients had difficulty in complying with ischemic post-conditioning and the final cohort comprised 40 patients underwent IPC. Their baseline characteristics are summarized in Table 1. The median age was 67 years (IQR, 59–71.8), and 29 patients (72.5%) were male. The median NIHSS score on admission was 13 (IQR, 11–16), and the median ASPECTS was 7 (IQR, 7–8). The most common occlusion site was the M1 segment of the MCA (30 patients, 75%), followed by tandem M1 and ICA occlusion (10 patients, 25%).

**Table 1:** Baseline characteristics of patients.

Characteristic	Total patients (*N* = 40)
Male sex, no. (%)	29 (72.5)
Age, median (IQR), year	67 (59–71.8)
NIHSS, median (IQR)	13 (11–16)
Occlusion site, no. (%)	
M1	29 (72.5)
M1 and ICA	10 (25)
ASPECT, median (IQR)	7 (7–8.8)
Vascular risk factor, no. (%)	
Hypertension	27 (67.5)
Diabetes mellitus	12 (30)
Dyslipidemia	31 (77.5)
Smoker	21 (52.5)
Interval between LKW and hospital arrival, median (IQR), h	4 (2–7)
Stroke etiology no. (%)	
LAA	33 (82.5)
CE	6 (15)
UD or other	1 (2.5)

TOAST consists of the following five groups: large artery atherosclerosis (LAA, ≥50% stenosis), cardioembolism (CE), small vessel occlusion (SVO), and other determined (OD) and undetermined etiologies (UD).

One patient (1/40, 2.5%) showed contrast extravagation; thus, we did not complete all circles (Supplementary Table 1). One patient with potential stenosis underwent IPC after angioplasty, and angiography showed thrombotic events after balloon temporary occlusion.

The difference between the M1 alone group and the M1 plus ICA group in the incidence of adverse events and technical facts is shown in Supplementary Table 1. In patients with M1 occlusion alone, the time from recanalization to the first balloon placement for IPC was numerically shorter than in those with tandem ICA occlusion. One patient showed contrast extravagation; thus, we did not complete all circles. One patient with potential stenosis underwent IPC after angioplasty, and angiography showed thrombotic events after balloon temporary occlusion. Two patients with tandem lesions, reocclusion of the target blood vessels occurred before IPC and they underwent thrombectomy again.

The difference between the LAA group and the non-LAA group in the incidence of adverse events and technical facts is shown in Supplementary Table 2. In patients with LAA the time from recanalization to the first balloon placement for IPC was shorter than that in patients without LAA. The first balloon deployment success rate was numerically higher in the LAA group compared with the non-LAA group, but no statistically significant difference was observed. In the LAA group, one patient suffered from thrombotic events. Two patients with potential ICAS had re-occlusion of the responsible vessel before IPC. The patient underwent additional balloon angioplasty before IPC.

## Discussion

Experimental studies have proven the neuroprotective effects of IPC, but there has not been any breakthrough in translational studies. The definition and clinical neurointerventional technique of IPC have not been comprehensively elaborated so far. Correct selection of patients and implementation of IPC can maximize its neuroprotective effects and prevent potential adverse events. Collateral circulation should be considered to completely block the blood flow. Due to extracranial sources of cerebral blood flow, interhemispheric collaterals, and variability in the anatomy of the circle of Willis, it is difficult to achieve complete occlusion of ICA (15, 16). Meanwhile, temporary occlusion of ICA rather than MCA can affect preoperative unobstructed blood vessels. Incomplete occlusion or redundant occlusion can both lead to differences and more uncertainties in the results. Therefore, in this study, we chose the middle cerebral artery as the site of IPC.

The procedure time for middle cerebral artery occlusion was significantly shorter compared with the ICA group, although there was no statistical difference. These findings suggest that the strategy targeting the middle cerebral artery was correct, and it may be simpler and easier for clinical application. At the same time, in cases with internal carotid artery occlusion, the risk did not significantly increase. The thrombotic events more frequently occurred in patients with simple middle cerebral artery occlusion compared with cases with tandem lesions. Patients with middle cerebral artery occlusion complicated with different segments of internal carotid artery occlusion were candidates for IPC.

Among all patients, one patient with potential stenosis underwent IPC after angioplasty, and angiography showed thrombotic events after dilated balloon filling. Considering the long procedure time, the pressurized irrigation fluid remained unchanged at the end of the surgery, and heparin saline irrigation was not performed. In the subsequent PROTECT series, we optimized the operation method. For patients who met the basic admission criteria, especially for patients with potential ICAS, we re-pressurized and administered heparin saline irrigation during the balloon procedure. No complications occurred in the subsequent PROTECT series.

Two patients with potential ICAS experienced the re-occlusion of the responsible blood vessel before IPC. We conducted the balloon catheter for IPC after balloon angioplasty forpatients. The underlying ICAS was considered to be refractory large vessel occlusion; thus, these adverse events could potentially increase the risk of IPC treatment in ICAS patients. Selecting patients with ICAS for IPC may have potential benefits or advantages. The prolonged duration of inflation has been used in many clinical trials to reduce the incidence of vascular dissection, risk of vascular rupture, and restenosis (17–19). The mechanism by which IPC may benefit such patients is complex. IPC may bring additional benefits because of longer balloon dilation time compared with conventional balloon angioplasty. It leads to plaque remodeling and modulates local coagulatory mechanisms, which may require specific targeted clinical research (20). Angioplasty can injure the inner lining of a blood vessel, which causes platelets to stick to the damaged area and form a blood clot. This clotting process appears to be a key factor in two main complications: acute vessel blockage (acute thrombotic occlusion) and the re-narrowing of the vessel over time (restenosis). IPC may reduce this damage to the vascular endothelium, possibly by moderating the balloon inflation strategy, thereby decreasing the coagulation response.

More importantly, the procedure time for IPC was shorter among patients with potential IAS. The perfusion injury may occur immediately after blood flow. Previous clinical studies conducted IPC for patients with ST-elevation myocardial infarction treated by primary PCI. They showed that IPC treatment might not be effective in patients who underwent thrombectomy before IPC compared with patients whose blood flow was partially restored by a guide wire or balloon catheter (21, 22). Longer operation time or complete reperfusion may offset the beneficial effects of IPC. The affected ICAS can show the location of the target lesion, which can help choose the location of temporary occlusion. As the same balloon catheter was used, the navigation of the balloon catheter was unnecessary, and the procedure time was shorter, facilitating IPC.

Our study has limitations. First, as a small-sample observational cohort study, the statistical power of our findings may be limited, but we guarantee the authenticity of all patient research information. Future research with larger sample sizes or randomized controlled trials is needed to further support these conclusions. Second, the number of patients in the LAA vs. no-LAA and M1 alone vs. M1 plus ICA subgroups was relatively small, which limited the statistical power and precluded definitive conclusions. The findings from these subgroup analyses should be considered exploratory and interpreted with caution.

To sum up, the incidence of adverse reactions following IPC was acceptable. As an improved reperfusion technique, it may potentially benefit patients with large vessel occlusion. In patients with ICAS, IPC technology may be particularly easier to implement and more likely to exert neuroprotective effects.

## Data Availability

The original contributions presented in the study are included in the article/Supplementary material, further inquiries can be directed to the corresponding author.
