# Correction: *In situ* post-ischemic conditioning by temporary balloon occlusion for acute ischemic stroke: a modified reperfusion technique and adverse event monitoring

**DOI:** 10.3389/fneur.2025.1722885

**Published:** 2025-10-27

**Authors:** Sifei Wang, Shuai Liu, Bohao Zhang, Xiao Jiang, Ming Wei, Hua Yan

**Affiliations:** ^1^Huanhu Hospital Affiliated to Tianjin Medical University, Tianjin Medical University, Tianjin, China; ^2^Tianjin Key Laboratory of Cerebral Blood Flow Reconstruction and Head and Neck Tumor New Technology Translation, Tianjin Neurosurgical Institute, Tianjin Huanhu Hospital, Tianjin, China; ^3^Department of Neurosurgery, Tianjin Huanhu Hospital, Tianjin Medical University, Tianjin, China

**Keywords:** modified reperfusion technique, acute ischemic stroke, large vessel occlusion, endovascular therapy, neuroprotective

In the published article authors Sifei Wang, Shuai Liu and Bohao Zhang were erroneously not acknowledged with equal contribution. The following statement has been added ‘†These authors have contributed equally to this work' and the superscript symbol added near their name.

The original version of this article has been updated.

