# Supplementary material for: In situ post-ischemic conditioning by temporary balloon occlusion for acute ischemic stroke: a modified reperfusion technique and adverse event monitoring
Source: Front Neurol. 2025 Oct 2;16:1634885. doi: 10.3389/fneur.2025.1634885 (PMC12527838; doi:10.3389/fneur.2025.1634885)
Supplement: Supplementary file 1 [file Table_3.DOCX]

eFigure1 IPC procedural timeline and balloon catheter placement strategy.





eFigure2 Flow chart gram.


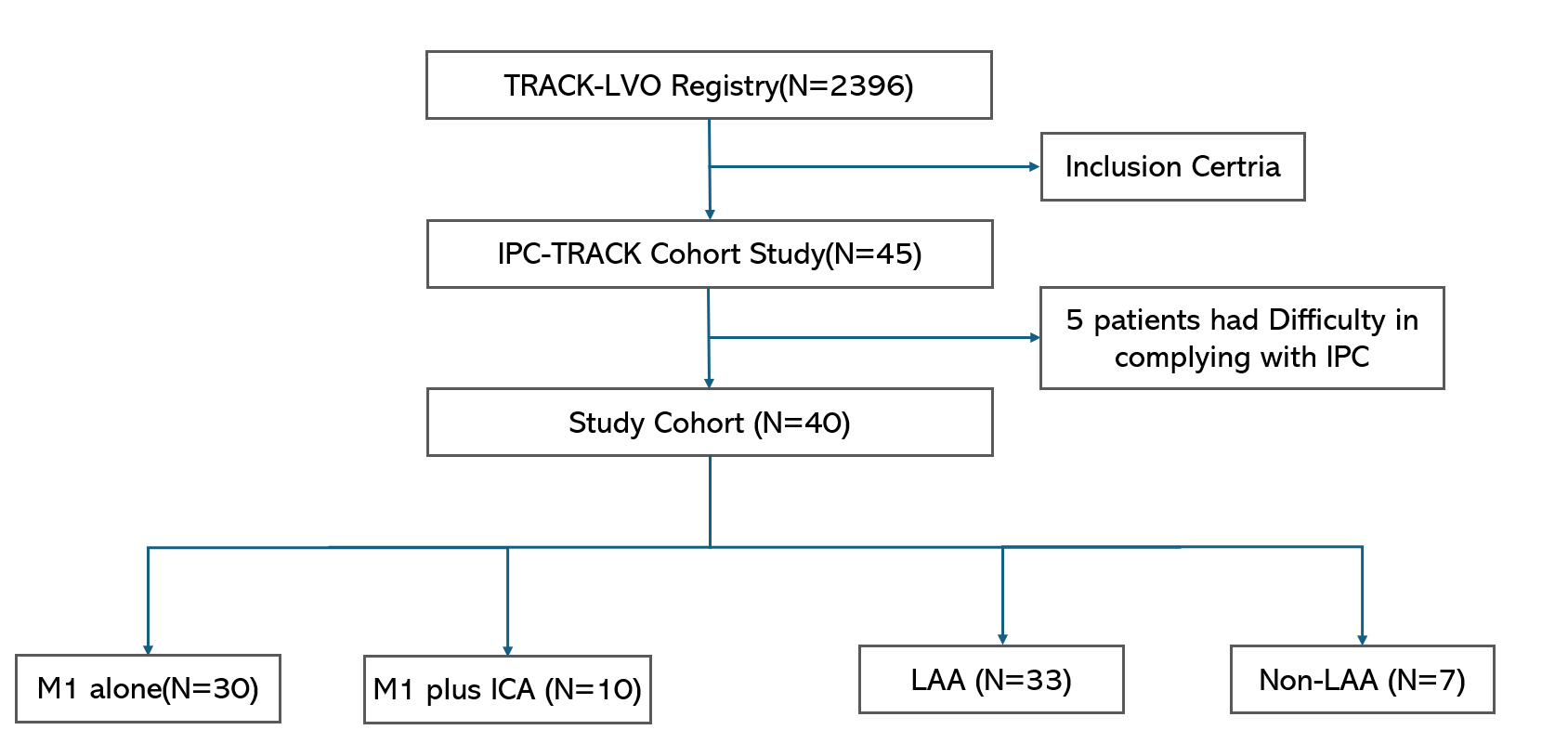


eTable 1 Technical facts and adverse events among M1 alone and M1 plus ICA group.

|  | M1alone（n=30） | M1 plus ICA （n=10） | P |
| --- | --- | --- | --- |
| Technical performance |  |  |  |
| Time from recanalization to the first balloon placement for IPC, min, median (IQR) | 10(1-22.25) | 19(10.75-36.25) | 0.064 |
| First balloon deployment success, No. (%) | 30(100) | 10(100) | 1 |
| Adverse events, No. (%) |  |  |  |
| Thrombotic event | 1(3.3) | 0 | 1 |
| Dissection | 0 | 0 | 1 |
| Contrast extravasation | 1(3.3) | 0 | 1 |
| Reocclusion before IPC | 2(6.7) | 0 | 1 |
| Reocclusion after IPC | 0 | 0 | 1 |
| Procedure-related mortality | 0 | 0 | 1 |
| Procedure-related morbidity | 0 | 0 | 1 |

|  | LAA（n=33） | non-LAA（n=7） | P |
| --- | --- | --- | --- |
| Technical performance |  |  |  |
| Time from recanalization to the first balloon placement for IPC  , min, median (IQR) | 10(1-18) | 28(10-40) | 0.049 |
| First balloon deployment success, No. (%) | 33(100) | 7(100) | 1 |
| Adverse events,No. (%) |  |  |  |
| Thrombotic event | 1(3) | 0 | 1 |
| Dissection | 0 | 0 |  |
| Contrast extravasation | 1(3) | 0 | 1 |
| Reocclusion before IPC | 2(6) | 0 | 1 |
| Reocclusion after IPC | 0 | 0 | 1 |
| Procedure-related mortality | 0 | 0 | 1 |
| Procedure-related morbidity | 1(3) | 0 | 1 |

eTable 2 Technical facts and adverse events among LAA and non-LAA groups
